# Assessment of Analgesic Efficacy of Bilateral Lumbar Erector Spinae Plane Block for Postoperative Pain following Lumbar Laminectomy: A Single-Blind, Randomized Clinical Trial

**DOI:** 10.1155/2023/5813798

**Published:** 2023-12-28

**Authors:** Seyed Amirreza Akhlagh, Arash Farbood, Mahsa Tahvili, Afshin Amini, Keyvan Eghbal, Naeimehossadat Asmarian, Mahsa Banifatemi, Seyed Ali Hosseini

**Affiliations:** ^1^Anesthesiology and Critical Care Research Center, Shiraz University of Medical Sciences, Shiraz, Iran; ^2^Department of Neurosurgery, Shiraz University of Medical Sciences, Shiraz, Iran; ^3^School of Medicine, Shiraz University of Medical Sciences, Shiraz, Iran

## Abstract

**Background:**

The erector spinae plane (ESP) block is a novel approach to minimizing postoperative pain. We investigated the efficacy and side effects of the ultrasonography-guided bilateral ESP block in reducing pain in the first 24 hours after lumbar laminectomy.

**Materials and Methods:**

We conducted a single-blind (statistical analyst and those responsible for recording patient information postoperation were unaware of the study groups) randomized clinical trial on 50 patients aged 18 to 65 with American Society of Anesthesiology (ASA) class I or II physical status scheduled for lumbar laminectomy surgery at Shahid Chamran Hospital, Shiraz, Iran. Patients were randomly allocated to the ESP block (26 participants) or control (24 participants) group. A bilateral ESP block was administered to patients in the first group before general anesthesia, which was provided identically to both groups. The postoperative time to the first request of analgesia, pain score, total opioid use, side effects, and patient satisfaction were compared between the groups.

**Results:**

Compared with the control group, patients in the ESP block group had significantly more postoperative pain relief in the first hour and until 24 hours (*P* < 0.05). The total opioid consumption was lower in the ESP block group (*P* < 0.001). However, the ESP block led to a higher rate of urinary retention (*P* = 0.008).

**Conclusion:**

The bilateral ESP block effectively reduces postoperative pain following lumbar laminectomy, minimizing the need for narcotics. Further research is needed to delineate ways to reduce urinary retention as its main complication. This trial is registered with IRCT20100127003213N6.

## 1. Introduction

Moderate to severe pain following surgery represents a major problem for patients, occurring in up to 80% of cases [1]. Regardless of its severity, postoperative pain may reduce patient satisfaction, delay mobility, and increase the risk of respiratory and cardiovascular complications (e.g., thromboembolism). Reduced postoperative pain has been associated with fewer complications, shorter hospital stays, and decreased deaths [2].

Laminectomy is a common surgery for patients suffering from spinal canal stenosis in which the lamina, the posterior part of the vertebra, is removed for pain and disability relief. Patients experience severe postoperative pain, usually within the first 12 hours. This pain is caused by postoperative irritation of the posterior horn neurons due to surgery-related tissue damage. Reducing postoperative pain decreases mortality, ensures early patient movement, and increases patient satisfaction [1–3].

While spinal anesthesia has replaced general anesthesia in many surgeries (e.g., caesarean section), regional blocks and fascial plain blocks feature fewer overall side effects and, in some cases, similar or even better analgesic effects. These procedures have therefore been proposed as reasonable alternatives to systemic narcotics and spinal anesthesia [4]. Regional anesthesia complements the role of multimodal anesthesia during surgery. Researchers seek to develop novel anesthesia methods to achieve a faster and easier procedure with fewer side effects and better outcomes [5], with a recent interest in fascial plane blocks as an alternative to regional blocks [6].

The erector spinae plane (ESP) block is a fascial plain block that can relieve neuropathic chest pain, postoperative pain, and posttraumatic pain. First described in 2016, this technique represents a safe, simple, promising, and well-performing alternative to the neuraxial block for various surgeries. In addition, this block reduces the risk of direct spinal cord injury, epidural hematoma, and central nervous system infection [7, 8]. The proposed mechanism behind this procedure is the blockage of the dorsal and anterior horns of the spinal nerves and sympathetic nerve fibers. Radiographic evidence suggests that the locally injected anesthesia is distributed up and down due to the expansion of the plane along the spine, facilitating successful anesthesia in the region of the neck, chest, abdomen, pelvis, and back in a variety of operations, including pyloroplasty, lipoma removal, breast reconstruction, inguinal hernia repair, hip reconstruction, cardiac surgery, and spine surgery [7–29]. Another important point regarding the ESP block is the use of low levels of anesthetics, which, as previous studies have shown, can block the pain-transmitting neurons without blocking other sensory and motor neurons [6]. This technique can also be used with a continuous catheter in the same area, inducing a long-term analgesic effect [7]. The analgesic effect of the ESP block has been demonstrated by the lack of narcotics required during and after surgery, along with the low pain scores reported by the patient in the early postoperative period [8].

Although many studies have been conducted on the ESP block for different types of surgery, the complications must be examined more closely. In addition, methods are needed to optimally test movement and sensory changes depending on the block's location [9]. Concerns like urinary retention as a potential complication underline the need for studies to optimize drug dosage, volume, and injection site. This study aimed to assess the efficacy of the bilateral ESP block with ultrasound guidance on reducing the severity of patients' pain during the first 24 hours after laminectomy, with particular consideration of urinary retention as a possible complication.

## 2. Patients and Methods

### 2.1. Study Design

This single-blind clinical trial was registered with the Iranian Registry of Clinical Trials (IRCT20100127003213N6) and approved by the Ethics Committee of Shiraz University of Medical Sciences (IR.SUMS.MED.REC.1398.047). We conducted this study at Shahid Chamran Hospital, affiliated with Shiraz University of Medical Sciences, Shiraz, Iran, from July to September 2019. The study included 50 patients aged 18 to 65 with American Society of Anesthesiologists (ASA) class I or II physical status who were candidates for lumbar laminectomy surgery.

### 2.2. Sample Size

The sample size of this single-blinded study was calculated based on the average effect size of 0.85 from a previous study [10] for the comparison of visual analog pain scores at postoperative time points between ESP block patients and controls, with a two-sided significance level of 0.05 and a drop-out rate of 15% at 24 hours. Ultimately, at least 52 patients were needed to achieve 80% power.

### 2.3. Eligibility Criteria

The inclusion criteria were as follows: lumbar laminectomy surgery candidates; age range of 18–65 years; ASA physical status I or II; and mental competency for cooperation and responding, including learning the numerical scoring criteria and working with a patient-controlled analgesia (PCA) pump. The patient's surgeon was made aware of his participation in the study and was allowed to perform alternative procedures if necessary. Exclusion criteria included a history of uncontrolled seizures, depression, local infection, rupture and repair of the dura during surgery, severe coagulation disorders, drug abuse or dependence, sensitivity to any of the compounds used in the study, need for mechanical ventilation for any reason after the operation, and patients with known psychosomatic disorders. The patients participated in this study after providing informed consent.

### 2.4. Randomization

Eligible patients were randomized using the block randomization method (https://www.sealedenvelope.com) and allocated to the treatment (ESP block) or control group in 13 blocks of size 4. The name of each patient's group was written and prepared in sealed envelopes by a single staff member who had access to the randomization list.

### 2.5. Intervention

After the patient entered the operating room, their basic information was recorded. The patient was then taught how to use the PCA pump. We asked the patients to describe their pain severity using a numerical rating scale (NRS), where zero indicated the absence of pain, and ten indicated the maximum possible pain.

The general anesthesia regime (including premedication, anesthesia induction drug, muscle relaxant, anesthesia maintenance, and analgesia with morphine sulfate 0.1 mg/kg) was identical in both groups. In the ESP block group, after gaining intravenous access and establishing standard monitoring, the patients were placed in a sitting position. Then, using a curved probe under ultrasound guidance (2 to 5 MHz), the transverse processes of the lumbar vertebrae were identified as close as possible to the surgical site, and 20 ml of 0.25% bupivacaine was injected just over the transverse process with a No. 22 spinal needle in the long axis view after making contact. The same procedure was repeated on the contralateral side. Patients in the control group did not receive this ESP block.

### 2.6. Primary Outcomes

In the postoperative recovery room, the time of the patient's first request for analgesics (from the time of operation termination) was recorded. After the patients retained awareness, the severity of pain was measured at intervals of 15, 30, 45, and 60 minutes according to the NRS. Pain was treated according to the following protocol: if the NRS score was less than 4, no action was taken; if it was 4 to 7, 1 mg of intravenous morphine was given every 5 minutes until achieving a score of below 4; and if the score was above 7, 2 mg of intravenous morphine was prescribed. The total amount of morphine consumed in the recovery room was recorded.

After transferring the patient to the ward, an intravenous PCA pump device loaded with a 20 ml syringe containing 0.5 mg/ml morphine solution was prepared with the following settings and connected to the patient's intravenous cannula: bolus dosage = 2 ml (1 mg); lockout intervals = 7 minutes; no continuous background infusion; and maximum injection dose over 4 hours = 60 ml (30 mg). In the ward, the patient's pain was assessed via the NRS and recorded once per hour in the first 6 hours, then every 2 h for the next 6 hours, and then every 4 h for the last 12 hours. During and between each pain assessment, the protocol above was followed, with additional intravenous morphine being administrated accordingly, and the prescribed morphine was recorded separately (PCA pump morphine and additional morphine). Data collection was performed by staff unaware of each patient's study group.

### 2.7. Secondary Outcomes

Postoperative pain control satisfaction level was evaluated on a five-point scale (1: completely dissatisfied; 2: dissatisfied, 3: neutral; 4: satisfied; 5: completely satisfied). Complications from morphine injections (itching, urinary retention, nausea, and vomiting) were assessed by a trained nurse every 4 h in the first 24 hours after surgery. Drug (morphine and bupivacaine) complications were recorded by a trained nurse at admission and every 4 hours; these complications were managed according to local protocols. During the study, if a patient was excluded, the case was recorded along with its reason (e.g., unplanned self-administration of narcotics/analgesics or loss of cooperation).

### 2.8. Statistical Analysis

Data were analyzed using SPSS software (version 22, SPSS Inc., Chicago, IL) and GraphPad software version 9. Continuous variables are presented as mean ± SEM, median (*Q*_1_–*Q*_3_), and categorical variables as numbers and percentages. Statistical analysis was done using the chi-squared or Fisher's exact test for categorical variables and the independent *t*-test or Mann–Whitney *U* test for continuous variables. Also, the repeated measures ANOVA test was used to analyze the significance of changes in data obtained over time. The accepted significance level was 0.05 or less, and the adjustment Bonferroni *P* value for multiple testing was 0.003.

## 3. Results

### 3.1. Study Participants

**Figure 1:**
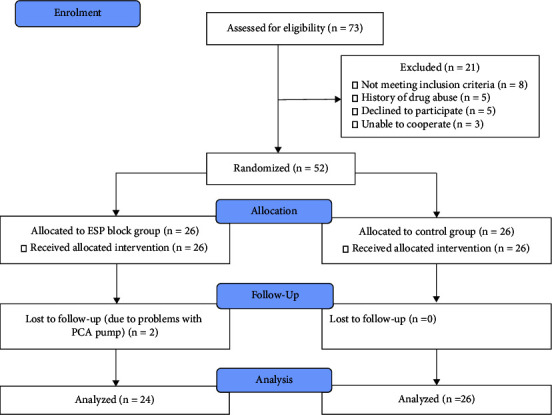
CONSORT flow diagram of the patient enrolment process.

Among the 73 eligible patients enrolled in the study, 21 were excluded, and two were lost to follow-up in the ESP block group. Finally, 50 patients completed the study, including 24 in the ESP block group and 26 in the control group (Figure 1).

The demographic data of the two groups are compared in Table 1. Significant differences were not observed between the ESP block and control groups regarding gender, age, and weight (*P* > 0.05).

### 3.2. Primary Outcomes

Table 2 compares the primary outcomes between the two groups. Patients in the ESP block group requested analgesic medication for the first time later than the control group (45 min vs. 22.5 min, *P* < 0.001). PCA consumption in the ward (mg), additional morphine consumption in the ward (mg), morphine consumed in recovery (mg), and total morphine consumption (mg) were lower in the ESP block group than in the control group. Also, the number of patients who requested additional morphine in the ward was significantly higher in the control group relative to the ESP block group (57.6% vs. 20.8%, *P*=0.008).

Based on repeated measures ANOVA, the time effect was significant (*P* < 0.001), the interaction between time and group was not significant (*P*=0.11), and the group effect was significant (*P* < 0.001) for pain scores (NRS) in the recovery room and ward. The adjusted Bonferroni *P* value for 16 points of time was 0.003.

**Figure 2:**
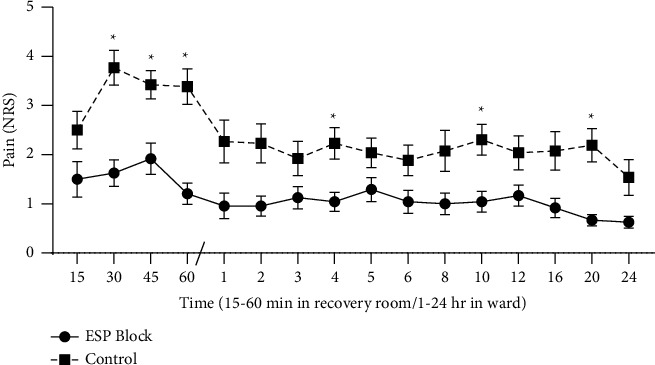
The trend of the changes in pain score on the numerical rating scale (NRS) in the recovery room and ward, compared between the erector spinae plane (ESP) block and control groups. Effect of time on NRS: *P* < 0.001; group effect: *P* < 0.001; group and time interaction effect: *P*=0.118. Recovery room *P* values: *t* = 15: *P*=0.064, *t* = 30: *P* < 0.001, *t* = 45: *P*=0.001, and *t* = 60: *P* < 0.001. Ward *P* values: *t* = 1: *P*=0.013, *t* = 2: *P*=0.007, *t* = 3: *P*=0.063, *t* = 4; *P*=0.003, *t* = 5; *P*=0.063, *t* = 6: *P*=0.036, *t* = 8: *P*=0.027, *t* = 10: *P*=0.002, *t* = 12: *P*=0.039, *t* = 16: *P*=0.012, *t* = 20: *P* < 0.001, and *t* = 24: *P*=0.024.

As Figure 2 shows, the pain scores (NRS) tended to be significantly higher in the control group in the recovery room and ward relative to the ESP block group. By performing multiple testing and considering the total adjusted *P* value (0.003), the *P* values at 30, 45, and 60 minutes in the recovery room, and the *P* values at 4, 10, and 20 hours in the ward, the control group had significantly higher pain scores than the ESP block group (Supplementary Table 1).

In the control group, the pain score increased with a sharper slope between 15 and 30 minutes compared with the ESP block group. The maximum pain score in the control group was nearly 4, compared with 2 in the ESP block group. After the time point of 30 min, the pain score in the control group decreased with a sharper slope compared with the ESP block group.

### 3.3. Secondary Outcomes

Regarding urinary retention caused by bupivacaine injection, there was a significant difference between the two groups (*P*=0.008). Six patients (25%) in the ESP block group had urinary retention, compared with none in the control group.

We observed no significant difference in patient satisfaction with the pain control method between the groups (*P*=0.27). The highest level of dissatisfaction was related to the control group (15%), while none of the patients were dissatisfied in the ESP block group. Also, 75% of patients were satisfied in the ESP block group, compared with 61% in the control group (Table 3).

## 4. Discussion

Although lumbar laminectomy is a common surgical and orthopedic technique for patients suffering from canal stenosis, suffering from severe pain in the first 12 hours after surgery is not uncommon. A multimodal pain control approach is often employed. The erector spinae plane (ESP) block is a simple-to-perform, reliable method for relieving postoperative pain. At the time of this investigation, this method had been rarely used to control postoperative pain following a lumbar laminectomy in a well-designed study. Hence, we investigated the efficacy of the ultrasonography-guided bilateral ESP block in reducing pain in the first 24 hours after lumbar laminectomy, demonstrating that postoperative pain scores (NRS) and morphine consumption were less in the ESP block group compared with the control group. Our findings indicate that the ESP block is superior to systemic opioids in many ways and can be a suitable alternative for controlling postoperative pain.

Regarding postoperative pain, our study demonstrated that patients who received the ESP block had significantly less pain intensity in the first hour and first day after surgery than controls; opioid use for pain relief was also less in the ESP block group. On the other hand, urinary retention was one of the most common postoperative complications among the patients receiving the ESP block, occurring significantly more commonly than in the control group. However, no difference was observed between the groups regarding other complications, such as nausea and vomiting. Although patients receiving the ESP block had higher overall postoperative satisfaction with pain control compared with the control group, the difference did not reach statistical significance. This may be due to fear of the block, needling before anesthesia, or misconceptions regarding postoperative pain in the ESP block group. Overall, the findings indicate the acceptable performance of the ESP block in controlling postoperative pain and reducing the need for intravenous opioids following lumbar laminectomy.

Forero et al. were the first to describe the ESP block in 2016. These researchers performed the block at the T5 level on two patients, demonstrating that this technique could be a good alternative for controlling neuropathic chest pain. The patients had almost no pain in the first 12 hours, agreeing with the results of the present study [7]. In 2019, Tsui et al. examined 242 articles on the ESP block and included 85 of them in their review [8]. Most studies used a single-dose injection method (80.2%), while the intermittent and continuous injection methods accounted for 12 and 7.9% of cases, respectively. In 90.9% of the studies, other pain relief methods were combined with the ESP block (multifaceted pain control). Interestingly, a reduction in narcotic use for pain relief was reported in 34.7% of the studies. Of the 85 studies, only two were randomized clinical trials (RCTs).

In 2020, another systemic review was published by Qiu et al., where the ESP block was done between the levels of T8 and L4 in 171 individuals. Decreased analgesic consumption and lower pain scores were reported, consistent with our study [11]. In 2022, a retrospective propensity score-matched study of 242 lumbar fusion patients was published by Soffin et al., in which patients in the ESP block group had less postoperative morphine consumption and hospital admission than the controls. Although pain scores were similar between the groups, postoperative nausea and vomiting (PONV) were less in the ESP block group. Their results regarding morphine requirements are comparable with our study, while we found differing results regarding PONV [12]. Huang et al. conducted a systemic review of 14 RCTs on patients undergoing thoracic and breast surgery, reporting lower pain scores and morphine usage with the ESP block relative to controls, as seen in our work [13].

In a systematic review published in 2021, Rizkalla et al. examined patients with spinal stenosis or spondylolisthesis undergoing decompression and lumbar spine surgery, concluding that the ESP block is a safe and effective method for postoperative pain relief with limited side effects [14]. In 2021, Broek et al. examined the addition of the ESP block to routine anesthesia care in posterior lumbar interbody fusion surgery candidates. Their results showed lower pain scores, less opioid usage via the PCA pump, and a shorter hospital stay in the ESP block group, consistent with our findings [15]. In 2020, Zhang et al. studied 60 patients undergoing open posterior lumbar surgery; compared to the control group, patients in the ESP block group (T12 level) needed less intraoperative sufentanil and postoperative morphine, had earlier ambulation times, and had higher modified observer assessment of alertness/sedation scores (MOAA/S) [16]. Among patients undergoing lumbar disc herniation surgery, Yorokoglu et al. found that although both the ESP block and control groups had similar pain scores (NRS), the ESP block group needed less morphine, agreeing with our study [17].

Kline et al. provided successful pain control for a single patient undergoing multi-level lumbar laminectomy using a modified dual-injection ESP block (11). This suggests that a modified ESP block may be required for more advanced multilevel surgeries, though RCTs must be conducted to obtain firm evidence. In a case series of six patients undergoing lumbosacral spine surgery, the ESP block at the lower thoracic levels provided effective postoperative pain control [6].

From 2018 to 2023, many studies have assessed the effectiveness of the ESP block in various surgeries in the thorax, abdomen, and pelvis, such as cardiac surgeries, hernia repairs, breast surgeries, cholecystectomy, and total pelvic reconstruction [9–29]. In all studies, consumption of opioids was less in the ESP block group (some during surgery and some in the postoperative period) than in the controls, with the pain intensity also being lower in most studies with the ESP block, which is consistent with our findings. Other reported advantages of the ESP block were better recovery quality, decreased need for mechanical ventilation, shorter ICU stay, and longer time to the first rescue analgesic dose.

In explaining the superior performance of the ESP block relative to systemic opioids, we examined the study of Kumar et al., who demonstrated that while tramadol reduced postlumbar laminectomy pain at rest more effectively compared with pregabalin, it had little effects on movement-induced pain. Hence, perhaps one explanation for the observed superiority of the ESP block over systemic narcotics in postoperative pain relief may be the control of movement-induced pain, for which opioids are less effective [1]. Also, Nagaraja et al. compared the ESP block with continuous thoracic epidural analgesia for cardiac surgery, declaring the ESP block as an acceptable way for postoperative pain control due to its safety and simplicity of performance [20].

Although our study and the prior literature confirm the efficacy of the ESP block in various surgeries, the side effects of this block have not been thoroughly investigated[30]. In this study, urinary retention was significantly more prevalent in the ESP block group than in the controls. This can be attributed to the side effects of morphine usage or the unpredictable distribution of the drug at the injection site, resulting in the unwanted block of the bladder nerves. Dautzenberg et al. demonstrated the unpredictable distribution of methylene blue after administering the ESP block to 11 cadavers [31]. Hence, it seems necessary to devise ways to minimize this significant complication of the ESP block.

### 4.1. Limitations

Performing the ESP block near the surgical site and informing patients about the block in such a way that it did not cause fear and anxiety before surgery were two challenges of this study. Despite our efforts in teaching the patients, the possibility of incorrect use of the PCA pump and underestimated or exaggerated reporting of pain on the NRS by patients should also be considered as limitations of this study. Finally, the lack of sufficient research to compare our study's findings regarding the ESP block's side effects against the literature should also be noted.

## 5. Conclusion

Our findings elucidate that the bilateral ESP block effectively reduces the postoperative pain of lumbar laminectomy patients, minimizing the need for narcotics and deterring their undesirable long-term effects. However, further research is needed to delineate ways to reduce the complications of the ESP block. Overall, we recommend the ESP block for use on a larger scale in the context of multifaceted pain control due to the simplicity of the procedure and the favorable outcomes.

## Figures and Tables

**Table 1:** Demographic data of the participants.

	ESP block (*n* = 24)	Control (*n* = 26)	*P* value
Sex, female	13 (54.2%)	17 (65.4%)	0.419
Age, years	47.12 ± 2.27	43.92 ± 2.73	0.375
Weight, kg	68.87 ± 1.78	69.81 ± 1.61	0.699

ESP: erector spinae plane. The chi-squared test or independent sample *t*-test was used. Values are presented as *n* (%) or mean ± SEM.

**Table 2:** Comparison of the use of pain relief medications between the erector spinae plane (ESP) block and control groups.

	ESP block (*n* = 24)	Control (*n* = 26)	*P* value
The first request for pain relief in recovery (min)	45 (30–52.5)	22.5 (15–30)	<0.001^*∗∗*^
PCA morphine used (mg)	3 (2–7)	12.5 (7–16)	<0.001^*∗∗*^
Additional morphine used in the ward (mg)	0 (0–0)	2 (0–3)	0.007^*∗*^
Morphine used in recovery (mg)	2 (0–4)	7 (6–9)	<0.001^*∗∗*^
Patients requesting additional morphine in the ward	5 (20.8%)	15 (57.6%)	0.008^*∗*^
Total morphine usage (mg)	4 (3–13)	20 (14–27)	<0.001^*∗∗*^

Values are shown as median (*Q*_1_–*Q*_3_) or frequency (percentage). The Mann–Whitney *U* test or chi-squared test was used. ^*∗*^*P* < 0.05; ^*∗∗*^*P* < 0.001.

**Table 3:** Comparison of patient satisfaction with the pain control method between the erector spinae plane (ESP) block and control groups.

	ESP block (*n* = 24)	Control (*n* = 26)	*P* value
Completely dissatisfied	0 (0)	0 (0)	0.27
Dissatisfied	0 (0)	4 (15.4)	
Neutral	3 (12.5)	3 (11.5)	
Satisfied	18 (75)	16 (61.5)	
Completely satisfied	3 (12.5)	3 (11.5)	

Values are shown as frequency (percentage). Fisher's exact test was used.

## Data Availability

All data used in this study are available upon request from Mahsa Tahvili (tahvili_m@yahoo.com).

## Supplementary Materials

Supplementary Materials All the descriptive information of the investigated variables in the study by group is shown in Supplementary Table 1.
