# Supplementary material for: Assessment of Analgesic Efficacy of Bilateral Lumbar Erector Spinae Plane Block for Postoperative Pain following Lumbar Laminectomy: A Single-Blind, Randomized Clinical Trial
Source: Pain Res Manag. 2023 Dec 28;2023:5813798. doi: 10.1155/2023/5813798 (PMC10766473; doi:10.1155/2023/5813798)
Supplement: Supplementary Materials — All the descriptive information of the investigated variables in the study by group is shown in Supplementary Table 1. [file 5813798.f1.docx]

**Supplementary 1**. Mean scores of the frequency of experience and noise level for each noise source (N = 148)

| Noise source | Frequency of experience | Noise level |
| --- | --- | --- |
|  | M±SD | M±SD |
| Human factor | | |
| Noise from emergency situations | 3.51±0.66 | 7.87±2.23 |
| The sound of evaluating the patient’s consciousness | 3.44±0.78 | 6.35±2.28 |
| Noise when the patient is hospitalized | 3.40±0.71 | 6.60±2.38 |
| Conversation of nurses, doctors, staff, and others | 3.36±0.75 | 5.94±2.12 |
| Patient’s high voice | 3.36±0.65 | 7.86±2.08 |
| The sound of nurses, doctors, and staff calling each other | 3.28±0.83 | 5.47±2.21 |
| Handover between nurses | 3.28±0.83 | 5.41±2.40 |
| The sound of the telephone conversation | 3.21±0.87 | 5.06±2.28 |
| The sound of tapping on a computer keyboard | 3.19±0.96 | 3.86±2.32 |
| Noise when moving the patient (exit, moving to the examination room, etc.) | 3.16±0.86 | 5.32±2.52 |
| The sound of opening and closing drawers or doors | 3.14±0.95 | 4.07±2.37 |
| Noise from various treatments required for patients | 3.14±0.90 | 4.48±2.34 |
| Moaning or crying of a patient | 3.13±0.70 | 6.97±2.50 |
| The sound of removing vinyl packing | 3.10±0.97 | 3.72±2.32 |
| Noise when the staff of various occupations visits | 3.07±0.83 | 4.85±2.26 |
| The laughter of nurses, doctors, and staff | 3.03±0.69 | 5.67±2.28 |
| The sound of greeting each other during the shift | 3.03±0.96 | 4.54±2.33 |
| Noise during medical rounds | 3.01±0.92 | 4.47±2.24 |
| The sound of the patient’s cough | 2.99±0.79 | 5.07±2.31 |
| The sound of medication preparation | 2.98±1.05 | 3.29±2.22 |
| Noise on deathbed | 2.98±0.90 | 5.36±2.74 |
| The sound of wearing a vinyl apron or gloves | 2.96±1.07 | 2.95±2.29 |
| Noise when performing the patient’s position change | 2.96±1.03 | 3.65±2.19 |
| Noise when educating patients or families | 2.89±0.80 | 4.65±2.12 |
| The sound of tidying things | 2.88±1.02 | 3.09±2.17 |
| The sound of cleaning the floor or surrounding environment | 2.87±1.05 | 2.90±2.22 |
| The sound of opening and closing the trash can | 2.85±1.01 | 3.20±2.32 |
| The footsteps of nurses, doctors, and staff | 2.80±1.00 | 3.93±2.46 |
| Noise when changing linen | 2.80±0.97 | 3.50±2.17 |
| Sound from medication administration | 2.80±1.05 | 3.08±2.11 |
| The sound of pulling out paper towels | 2.74±1.08 | 2.42±2.13 |
| Sound from washing medical devices | 2.70±1.03 | 3.02±2.33 |
| Noise when bathing a patient in bed | 2.46±1.10 | 2.98±2.23 |
| The sound of the patient snoring | 2.42±0.81 | 4.05±2.33 |
| The sound of patients talking to their families during visiting hours | 2.21±0.87 | 3.55±2.37 |
|  | 3.00±0.63 | 4.55±1.54 |
| Medical device factor | | |
| Various alarm sounds | 3.73±0.53 | 7.76±2.07 |
| Various bell sounds | 3.55±0.65 | 6.79±2.30 |
| Sound of suction device operation | 3.49±0.73 | 6.28±2.30 |
| Sound of nebulizer operation | 3.47±0.71 | 6.28±2.54 |
| Sound of dragging medical equipment, car, bed, or chair | 3.28±0.88 | 5.11±2.50 |
| Operating sound of the barcode printing machine | 3.22±0.90 | 4.70±2.39 |
| Sound of installing infusion pump | 3.12±0.93 | 4.76±2.46 |
|  | 3.41±0.62 | 5.96±1.87 |
| Environmental factor | | |
| Sound of an announcement | 3.06±0.80 | 5.29±2.37 |
| Sound of water in the sink | 2.97±1.05 | 3.07±2.21 |
| Sound of air conditioner or ventilation | 2.89±1.00 | 3.93±2.50 |
|  | 2.97±0.83 | 4.74±1.55 |
| Total | 3.06±0.62 | 4.74±1.55 |
